# Exercise Timing Matters for Glycogen Metabolism and Accumulated Fat Oxidation over 24 h

**DOI:** 10.3390/nu15051109

**Published:** 2023-02-23

**Authors:** Kaito Iwayama, Jaehoon Seol, Kumpei Tokuyama

**Affiliations:** 1 Faculty of Budo and Sport Studies, Tenri University, Nara 632-0071, Japan; 2 International Institute for Integrative Sleep Medicine (WPI-IIIS), University of Tsukuba, Ibaraki 305-8575, Japan

**Keywords:** postprandial state, postabsorptive state, whole-room indirect calorimeter, glycogen

## Abstract

Due to increasingly diverse lifestyles, exercise timings vary between individuals: before breakfast, in the afternoon, or in the evening. The endocrine and autonomic nervous systems, which are associated with metabolic responses to exercise, show diurnal variations. Moreover, physiological responses to exercise differ depending on the timing of the exercise. The postabsorptive state is associated with greater fat oxidation during exercise compared to the postprandial state. The increase in energy expenditure persists during the post-exercise period, known as “Excess Post-exercise Oxygen Consumption”. A 24 h evaluation of accumulated energy expenditure and substrate oxidation is required to discuss the role of exercise in weight control. Using a whole-room indirect calorimeter, researchers revealed that exercise performed during the postabsorptive state, but not during the postprandial state, increased accumulated fat oxidation over 24 h. The time course of the carbohydrate pool, as estimated by indirect calorimetry, suggests that glycogen depletion after postabsorptive exercise underlies an increase in accumulated fat oxidation over 24 h. Subsequent studies using ^13^C magnetic resonance spectroscopy confirmed that the variations in muscle and liver glycogen caused by postabsorptive or postprandial exercise were consistent with indirect calorimetry data. These findings suggest that postabsorptive exercise alone effectively increases 24 h fat oxidation.

## 1. Introduction

**Figure 1:**
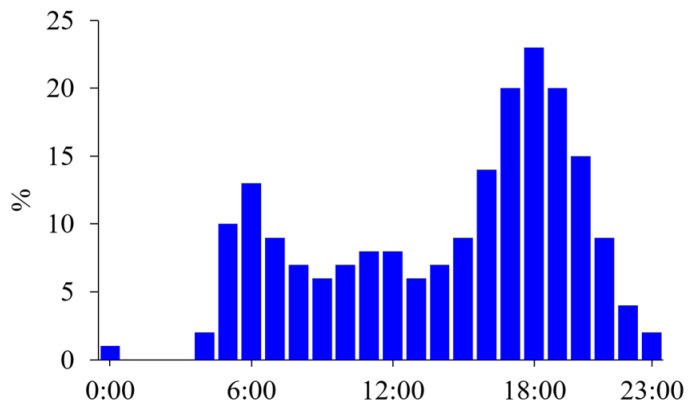
Time use survey on sports, exercise, and recreational activities. Diurnal variations in the percentage (%) of people participating in sports, exercising, or performing recreational activities are shown in 1-h intervals based on the time use survey in a working day in the United States [1]. People mainly exercised after their working hours, but some exercise in the morning, probably before breakfast.

According to surveys on time use conducted in the United States [1] and Japan [2], more people exercise in the postprandial state in the late afternoon, and few individuals exercise in the postabsorptive state before breakfast (Figure 1). Additionally, most individuals choose to exercise when it best fits their schedule; thus, there are variations in exercise timing among individuals. In professional athletes, daily training is frequently divided into multiple sessions. A recent survey found that 48% of elite endurance athletes reported performing at least some training sessions in the morning in the postabsorptive state [3]. The most common reasons for this included weight loss or body composition goals.

Regular exercise helps to achieve and maintain the desired body composition [4] and reduces the risk of multiple chronic health conditions [5]. Specific recommendations regarding the duration and intensity of exercise necessary to maintain a healthy body are provided by the World Health Organization physical activity guidelines: 150–300 or 75–150 min of moderate- or vigorous-intensity physical activity per week, respectively, or an equivalent combination of moderate- and vigorous-intensity aerobic physical activities per week [6]. The physical Activity Guidelines for Americans also suggest that exercise should be performed at least three days a week to avoid excessive fatigue and an increased risk of injury [7]. However, the preferable time (morning, afternoon, or evening) or nutritional state (postabsorptive or postprandial) when exercise is performed is not specified in the guidelines. The endocrine and autonomic nervous systems associated with metabolic responses to exercise exhibit diurnal variations [8,9]. Moreover, physiological responses to exercise may differ depending on the timing of the exercise. Shibata et al. recently proposed the term “chrono-exercise,” which associates exercise effectiveness with the time when it is performed [10]. Various studies have examined how exercise timing relates to food intake [11], circadian rhythm [12,13], energy substrates [14], body composition [15,16,17,18], training adaptations [19,20], and glucose metabolism [21,22]. Taken together, it appears that there may be a best time to exercise.

The evaluation of the accumulated energy expenditure and substrate oxidation over 24 h is essential in order to discuss the role of exercise on body weight control. The mechanisms underlying the time or nutritional state-specific effects of exercise on 24 h energy metabolism are currently being studied. In the present review, we discussed the effects of exercise timing on energy metabolism, particularly on fat oxidation. First, fat oxidation during different exercise protocols was compared using indirect calorimetry with a face mask or mouthpiece. Second, accumulated fat oxidation over 24 h was compared using indirect calorimetry in a whole-room metabolic chamber. Third, the time course of glycogen content in the liver and muscles was determined by conducting a ^13^C magnetic resonance spectroscopy (MRS) study. The ^13^C MRS confirmed our indirect calorimetry-based hypothesis that exercise timing affects diurnal glycogen content in the body.

## 2. Effects of Exercise Timing on Fat Oxidation during Exercise

If the motive of exercise is to reduce body fat, increasing fat oxidation relative to energy expended is critical [19]. Hence, understanding the factors that increase or decrease fat oxidation is key. Exercise intensity is one of the most important determinants affecting fat oxidation during exercise. The relative contribution of fat oxidation to total energy expenditure is greater at lower exercise intensities, and the energy is primarily obtained from the oxidation of free fatty acids in plasma [23]. Additionally, the rate of fat oxidation is increased in low-to-moderate intensity exercise, whereas it is decreased with high-intensity exercise [23]. Therefore, low-intensity exercise rather than high-intensity exercise is recommended to maximize fat oxidation and fat loss.

Fat oxidation during exercise has also been reported to vary depending on the timing and intensity of the exercise. According to Amaro-Gahate et al. [24], fat oxidation during graded exercise was greater between 5 and 8 PM than between 8 and 11 AM. Similarly, Sharma and Agarwal. [25] discovered that fat oxidation during steady-state exercise was higher between 3 and 4 PM than between 9 and 10 AM. Furthermore, serum-free fatty acid levels were significantly higher at 2 h after 60 min of steady-state exercise in the evening (between 5 and 6 PM) than in the morning (between 9 and 10 AM) [26]. To increase fat oxidation, evening exercise has been suggested over morning exercise [27]. Although these studies were conducted under standardized dietary conditions prior to experimental exercise conditions, the relative time between exercise and eating in real life is not fixed. The physical response to exercise is affected not only by the time of day but also by the nutritional status, which can be postprandial or postabsorptive. Particularly, the morning after an overnight fast, which lasts 8–10 h after the last meal, is a characteristic time of the day to spare carbohydrates and increase reliance on fat as a substrate for energy supply [28]. The only time the postabsorptive state occurs in a typical three-meal-a-day lifestyle is before breakfast. Exercise performed in the postabsorptive state is associated with greater fat oxidation than in the postprandial state [29,30].

**Figure 2:**
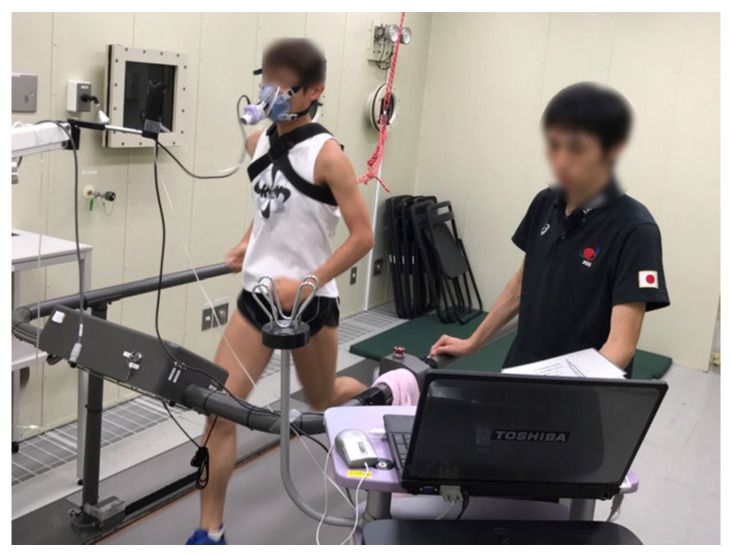
Indirect calorimetry using a face mask. Expired air was collected through a face mask, limiting the measurement duration to a few hours. The expired air was subjected to analysis of O_2_ and CO_2_ concentration.

Based on previous findings, it is possible to determine the optimal exercise conditions for losing body fat. However, the increase in whole-body fat oxidation persists not only during exercise but also after exercise [31]. Moreover, the intensity of exercise may affect post-exercise nutrient oxidation differently. Indeed, when high-intensity exercise is compared to low-intensity exercise, there is evidence of a greater reliance on energy from fat during the post-exercise period [32]. As a result, the energy source after high-intensity exercise shifts to fat-dominant sources; total fat oxidation during exercise remains constant, and a 3 h recovery period is unaffected by exercise intensity [33]. Therefore, to completely understand the implications of exercise for body weight regulation, the long-term effects of exercise on nutrient oxidation must be considered. However, assessing energy metabolism over an extended period, including eating and sleeping, would be difficult with a method that completely covers the mouth with a mask (Figure 2).

## 3. Effects of Exercise Timing on 24 h Fat Oxidation: Whole-Room Metabolic Chamber Study

**Figure 3:**
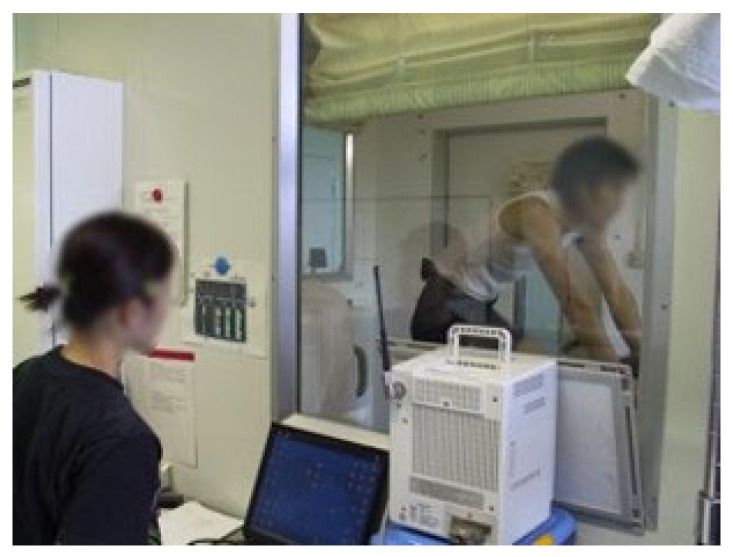
Whole-room indirect calorimetry. Changes in O_2_ and CO_2_ concentrations of the entire chamber are measured to assess O_2_ uptake and CO_2_ production of the participants without a mask and mouthpiece. This system, also known as a human calorimeter, allows us to track energy metabolism over time, including during sleep [37]. This method has limitations in assessing dynamic changes in energy metabolism caused by the dilution of the expired air with chamber air. Hence, efforts have been made to improve time resolution of the system [38].

Several studies have been conducted to examine 24 h metabolism under comparable energy expenditure during exercise to investigate the effects of different exercise intensities on total fat oxidation using whole-room indirect calorimetry (Figure 3). The increase in energy expenditure persists during the post-exercise period, known as “Excess Post-exercise Oxygen Consumption” [34,35,36]. Moreover, several studies have assessed energy metabolism under an energy-balanced condition, i.e., energy intake and expenditure over 24 h are balanced to assess accumulated fat oxidation for over 24 h, including during the post-exercise period. Surprisingly, there is a consensus in the literature that postprandial exercise does not increase 24 h fat oxidation in an energy-balanced condition; exercise was accompanied by an increase in energy intake, as planned in the experimental design, because over- and under-feeding had profound effects on nutrient oxidation (Table 1).

A study from Colorado, United States, compared fat oxidation in male and female participants in three trials: non-exercise control and exercise at 40% or 70% VO_2max_. Exercise began at 10 AM and lasted approximately 100 and 60 min for low- and high-intensity exercise, respectively, to burn 400 kcal. In the trial with exercise, participants consumed extra calories to maintain an energy-balanced condition. Low-intensity exercise increased fat oxidation during exercise, but 24 h fat oxidation among the three conditions, including in sedentary controls, was similar [39]. According to this study, exercise intensity as well as exercise per se does not influence accumulated fat oxidation over 24 h. Therefore, exercise does not significantly increase 24 h fat oxidation, and the belief that exercise contributes to increased fat oxidation may need to be reconsidered [40]. These studies indicate that the effects of exercise on energy metabolism cannot be determined solely through exercise alone but must be assessed over a longer period of time, including during post-exercise.

**Table 1:** Effects of exercise on accumulated fat oxidation over 24 h under an energy-balanced condition.

Participants	Exercise		Effect of Intensity	Effect of Exercise	Ref.
	Intensity	Duration			
Lean female	50%VO_2max_	1 h	NS		[41]
	100% VO_2max_	2 min × 15 trials			
Obese male	38%W_max_	1 h	NS		[42]
	80/50% W_max_	6 sets of 2.5 min each			
Lean female and male	No exercise		NS	NS	[39]
	40% VO_2max_	100 min			
	70% VO_2max_	60 min			
Obese female and male	No exercise		NS	NS	[43]
	40% VO_2max_	60 min			
	70% VO_2max_	30 min			
Lean female and male	No exercise		NS	NS	
	40% VO_2max_	60 min			
	70% VO_2max_	30 min			
Young male	No exercise			NS	[44]
	60% VO_2max_	300 kcal			
Old male	No exercise			NS	
	60% VO_2max_	300 kcal			
Lean sedentary male	55%VO_2max_	60 min		NS	[40]
Lean trained male	55%VO_2max_	60 min			
Obese sedentary male	55%VO_2max_	60 min			
Male	50%VO_2max_	60 min		NS	[45]

NS; not significant.

**Figure 4:**
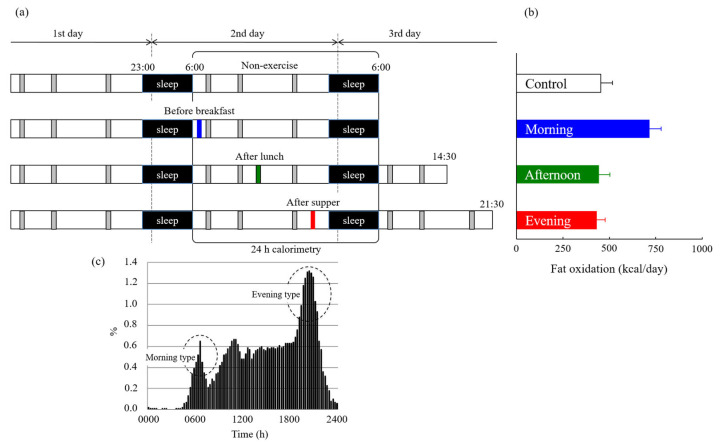
(**a**) Experimental protocol of our study [46]. Indirect calorimetry using a whole-room metabolic chamber was performed in 10 young non-obese men over 24 h. Participants remained sedentary (control) or performed 60 min of exercise at 50% VO_2max_ before breakfast (morning), after lunch (afternoon), or after supper (evening). All trials were designed to be performed in an energy-balanced state over 24 h. (**b**) Accumulated fat oxidation over 24 h following postprandial exercise was not higher than that in sedentary conditions. Only exercise before breakfast increased 24 h fat oxidation more than that in sedentary conditions. (**c**) Comparison of exercise timing in the study of diverse time use for sports, exercise, and recreational activities among Japanese [2].

Incorporating post-exercise evaluations may provide new insights into the effects of the pre-exercise nutritional status on fat oxidation. No studies have assessed the effects of exercise performed in a postabsorptive state on 24 h fat oxidation in an energy-balanced condition when we began our series of experiments. We compared 24 h fat oxidation in four trials that included a sedentary condition (non-exercise controls) and exercise in the morning (postabsorptive), afternoon, or evening (postprandial), to assess the accumulation of postabsorptive or postprandial exercise-induced fat oxidation [46]. Experimental exercise started at 6:30 AM, 2:30 PM, and 8:30 PM for 60 min at 50% VO_2max_. All trials were designed to be under an energy-balanced state for over 24 h. Energy expenditure during 60 min of exercise was similar among the three exercise trials (morning, 525 ± 28; afternoon, 527 ± 27; evening, 529 ± 27 kcal/60 min). However, fat oxidation during exercise was higher in the morning trials (158 ± 13 kcal/60 min) than that in the afternoon (42 ± 7 kcal/60 min) and evening trials (42 ± 5 kcal/60 min). Compared to the two postprandial exercises, postabsorptive exercise resulted in not only greater fat oxidation during exercise but also in increased fat oxidation over 24 h (Figure 4). Furthermore, compared to non-exercise controls, 24 h fat oxidation increased following exercise in the postabsorptive state, whereas this was not observed after postprandial exercise. Notably, urinary nitrogen excretion over 24 h, which is a marker of protein oxidation, was not significantly different between the four trials (morning, 311 ± 25; afternoon, 365 ± 28; evening, 340 ± 24; control; 317 ± 38, kcal/day). This study advocated that the increase in fat oxidation due to postabsorptive exercise was not offset in 24 h. Similar studies adopting different exercise intensities or timing similarly reported that postabsorptive exercise increases fat oxidation [47,48,49,50]. Furthermore, our findings in male participants were also confirmed in female participants [51]. This series of studies was conducted with a standardized diet and limited the use of supplements on the day before the experiment. Additionally, the participants ranged from healthy non-athletes to endurance athletes. Thus, these studies suggest that the postabsorptive state is the only spontaneous time of the day that increases fat oxidation following exercise and under an energy-balanced condition.

**Figure 5:**
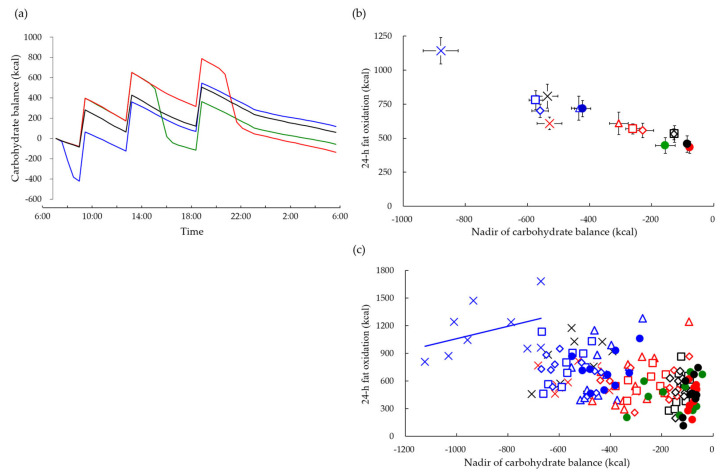
Carbohydrate balance and 24 h fat oxidation. (**a**) Carbohydrate balance relative to the start of 24 h calorimetry was estimated as the difference between input (meal consumption) and output (carbohydrate oxidation) for control (**―**), morning exercise (**―**), afternoon exercise (**―**), and evening exercise (**―**) [46]. (**b**) Relationship between nadir of carbohydrate balance and 24 h fat oxidation for control (●), morning exercise (●), afternoon exercise (●), and evening exercise (●) [46]. Data from our previous studies were also plotted: 12 males completed two trials of 60 min of exercise at 50% VO_2max_ before (▵) or after (▵) breakfast [47], 9 males completed three trials of 100 min of exercise at 65% VO_2max_ before breakfast (×) or after lunch (×), and 50 min each before breakfast and after lunch (×) [48], 10 males completed three trials of 60 min of exercise at 60% VO_2max_ before breakfast (□) and after lunch (□) or non-exercise control (□) [49], 11 males completed three trials of 60 min at 60% VO_2max_ before breakfast (◊) and after lunch (◊) or non-exercise control (◊) [50]. The correlation coefficient was −0.44 (*n* = 154; *p* < 0.01). (**c**) Criteria of nested data (i.e., 0.1 < ICC), namely the intraclass correlation coefficients (ICC) of transient carbohydrate deficits, and 24 h fat oxidation were 0.87 and 0.39, respectively. Transient carbohydrate deficits exhibited significant a negative association with 24 h fat oxidation in between experiments (B = −0.73, 95% CI: −0.87, −0.58). However, within the experiment, transient carbohydrate deficits show a significant positive association with 24 h fat oxidation (B = 1.35, 95% CI: 0.94, 1.76) [52]. As an example, the regression line is shown only for the trial in which 100 min of exercise at 65% VO_2max_ was performed before breakfast.

The above studies measured macronutrient oxidation and energy expenditure using a room-size metabolic chamber and 24 h indirect calorimetry. The main advantage of this method is that it can estimate the time course of energy and nutrient balance relative to the beginning of 24 h calorimetry as the difference between the input (meal consumption) and output (oxidation). The lowest values of relative carbohydrate balance observed through the time courses, i.e., greatest transient energy and carbohydrate deficit, were greater following postabsorptive exercise than postprandial exercise (Figure 5a). Combined data from five studies revealed a negative correlation between transient carbohydrate deficit and 24 h fat oxidation (Figure 5b). The magnitude of transient carbohydrate deficit, i.e., the lowest relative carbohydrate balance value, may indicate the amount of glycogen stored as a source of carbohydrate energy. A comparison of the group mean, which reflects differences in experimental conditions, suggested that the more glycogen was depleted, the more fat was oxidized over 24 h. However, we recognize that the data included a hierarchical structure, called nested data. A comparison of subjects within each experimental group revealed a positive correlation between relative carbohydrate deficit and 24 h fat oxidation, suggesting that the less that glycogen was depleted during exercise, the more that fat was oxidized over 24 h. This positive correlation reflects individual differences in fat oxidation, i.e., subjects with less depletion of glycogen stores oxidized more fat during exercise and over 24 h, reflecting individual differences in fat oxidation capacity such as muscle fiber type and mitochondrial density (Figure 5c).

**Figure 6:**
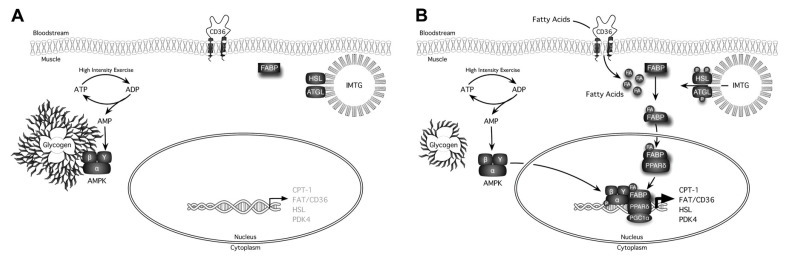
Graphic representation of some of the signaling events that are differentially activated by exercise in either a high-glycogen (**A**) or low-glycogen state (**B**) [56]. FA; fatty acid, PDK4; pyruvate dehydrogenase kinase 4, ATGL; adipose triglyceride lipase, IMTG; intramuscular triglyceride, FABP; FA-binding protein, PPAR; proliferator-activated receptor, PGC-1α; PPARγ coactivator-1α.

These results of indirect calorimetry suggest a potential mechanism by which diurnal changes in glycogen levels influence 24 h fat oxidation. Substrate oxidation shifts from carbohydrate to fatty acid during overnight fasting [53], and hormonal changes activate several transcription factors as well as modulate gene expression [54]. One possible physiological explanation for these findings is that variability in glycogen level plays a role in whole-body energy metabolism. Liver and muscle glycogen is a source of energy, and its quantity influences whole-body energy metabolism. Specifically, depletion of glycogen in the liver stimulates lipolysis in adipose tissues through a central nervous system-mediated mechanism [55], and a reduced muscle glycogen level locally triggers sequential events, including the weakened interaction of AMP-activated protein kinase (AMPK) with glycogen, enhanced AMPK activity, altered intracellular localization of AMPK, and upregulated expression of genes associated with fat oxidation such as carnitine palmitoyltransferase (CPT-1), fatty acid translocase (FAT/CD36), and hormone-sensitive lipase (HSL) (Figure 6) [56]. Hence, glycogen in the liver and muscle plays a role as an energy storage site but also a regulator of whole-body energy metabolism. Based on these findings, the underlying mechanisms of exercise performed in a postabsorptive state that increases 24 h fat oxidation seem to be due to the enhanced availability of free fatty acids and their oxidative capacity as a result of the transient glycogen deficit. Nevertheless, no previous studies have reported fluctuations in liver and muscle glycogen levels following postabsorptive or postprandial exercise.

## 4. Effects of Exercise Timing on Glycogen Metabolism: ^13^C MRS Study

**Figure 7:**
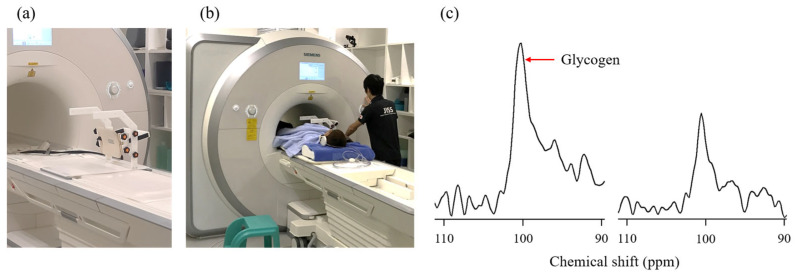
Muscle and liver glycogen measurements. (**a**) ^13^C-^1^H double-tuned surface coil measuring 10 cm used for glycogen measurement. We used the same coils for both the muscle and liver. (**b**) View of ^13^C magnetic resonance spectroscopy measurement of the liver. When measuring muscle glycogen levels, the coil was placed near the area concerned. (**c**) Example of muscle ^13^C spectra pre-exercise (left) and post-exercise (right).

**Figure 8:**
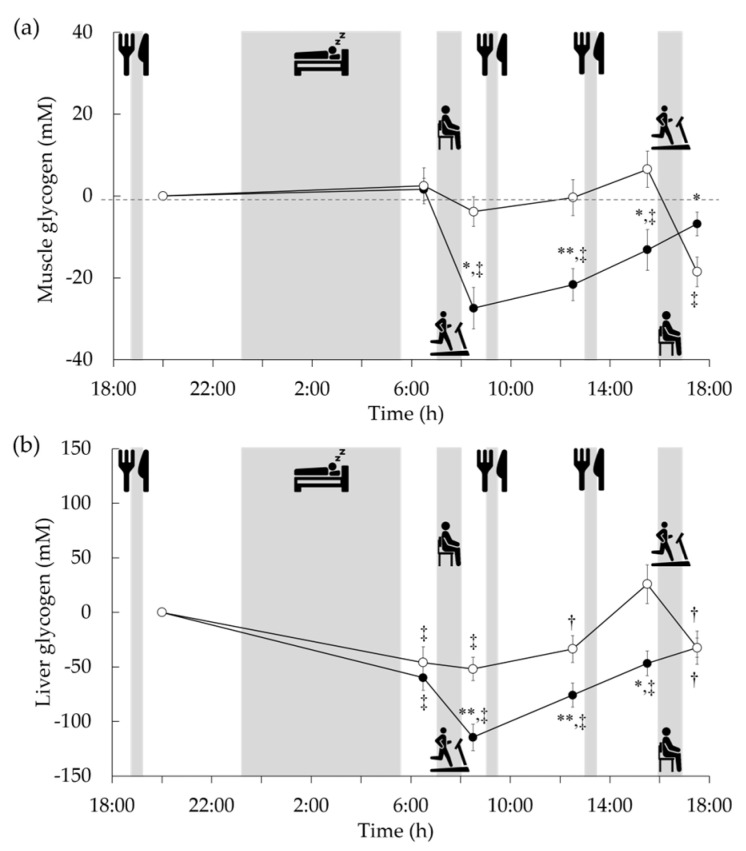
Changes from baseline (after supper, before bedtime) in muscle (**a**) and liver (**b**) glycogen stores after exercise in postabsorptive (closed circle) and postprandial (open circle) conditions [60]. Values are shown as mean ± standard error for the nine participants. * *p* < 0.05 vs. postprandial trial. ** *p* < 0.01 vs. postprandial trial. † *p* < 0.05 vs. baseline. ‡ *p* < 0.01 vs. baseline.

Glycogen has been traditionally assessed in vivo using biopsy wherein a piece of tissue is removed, in this case, from the liver and muscles. This is a difficult and invasive procedure and is associated with the infrequent incidence of side effects, especially in the liver [57]. As a result, diurnal variation analysis of glycogen has been restricted owing to the discomfort to participants and limitation in the number of measurements. Recent developments in ^13^C MRS have permitted the use of non-invasive techniques to assess the glycogen content in human skeletal muscles and liver. ^13^C MRS can be used to evaluate the glycogen content in different tissues several times a day without causing much inconvenience to the participants. ^13^C MRS was performed using a clinical 3 Tesla superconducting MR scanner as described [58,59]. Briefly, ^13^C spectra were collected with a ^13^C-^1^H double-tuned diameter surface coil set on the muscle or the right-hand side of the trunk (by the liver). The ^13^C spectrum of muscle was collected with a repetition time of 200 ms and 4500 acquisitions, requiring 15 min per measurement. Moreover, the liver ^13^C spectrum was collected with a repetition time of 160 ms and 6000 acquisitions, requiring 16 min per measurement. Since the glycogen spectrum was fully decoupled, as shown in Figure 7c, curve fitting was performed assuming that the glycogen peak was a singlet with a chemical shift of 100.5 ppm. Glycogen contents were determined by comparing the ^13^C spectra of muscle and liver with that of external standard solutions (muscle: 150 mM glycogen from oysters and 50 mM KCl, liver: 200 mM glycogen from oysters and 150 mM KCl). We examined glycogen variability in sedentary participants using this technique [59]. In the absence of specific exercise, glycogen content in the calf fluctuated little throughout the day, whereas glycogen in the liver fluctuated greatly, with the lowest level after an overnight fast. Furthermore, we examined the diurnal variation in glycogen by combining these findings with the timing of exercise either after an overnight fast or in the afternoon [60]. Glycogen content in the liver, which had been lowered by overnight fasting, was reduced to about half the level of the previous night following 60 min of exercise before breakfast. Subsequently, liver glycogen remained significantly lower than the previous night even after breakfast and lunch (Figure 8). In contrast, due to the preceding replenishment by breakfast and lunch, afternoon exercise did not result in a relatively large decrease in liver glycogen content compared to the previous night. These diurnal variations are similar to the carbohydrate balances estimated by indirect calorimetry (Figure 5a). However, our ^13^C MRS study did not measure the effects of supper and sleep on the variability of post-exercise liver and muscle glycogen content. Following afternoon exercise, the liver glycogen content was similar, but the muscle glycogen content was lower than that during exercise in a postabsorptive state. Thus, the relationship between 24 h fat oxidation and glycogen variability remains unclear. Since glycogen variation during the day has been demonstrated, it has been suggested that the part of the mechanism by which exercise performed in a postabsorptive state increases 24 h fat oxidation may be due to a transient muscle and liver glycogen deficiency.

## 5. Limitations and Future Perspective

Our studies on accumulated 24 h fat oxidation and glycogen content using whole-room indirect calorimetry and ^13^C MRS assessed the effect of a single session of exercise performed in a postabsorptive or postprandial condition [46,47,48,49,50,51,58,59]. These results suggest that exercise performed in the postabsorptive state increases fat oxidation due to transiently reduced glycogen levels, particularly in the liver.

It may be necessary to consider the relative time between exercises and meals to optimize an individual’s health through increased fat oxidation. Although the theoretical evidence is clear, there is no agreement on whether postabsorptive exercise results in greater long-term fat loss than postprandial exercise. Arciero et al. [61] found that 12 weeks of training that included exercise after an overnight fast reduced abdominal fat in female participants. However, several other studies have reported that postabsorptive exercise for 4 [18] or 6 [16,62] weeks did not show significant differences in body fat percentage or body fat mass compared to postprandial exercise.

In our previous study, the increase in fat oxidation following 60 min of postabsorptive exercise compared to postprandial exercise amounted to approximately 12 g [46]. Even with a completely controlled diet and performance of this exercise three days a week for 6 weeks, the calculated excess of fat oxidation was only 216 g. Although the benefits of continuous exercise are not limited to acute increases in fat oxidation, meaningful changes in body fat mass take time. More importantly, under the same conditions of energy and macronutrient intake conditions, greater fat oxidation over 24 h implies more carbohydrate storage. Of all the energy sources in the body, glycogen has a very low storage potential compared to fat and protein [63]. Thus, because of the limited glycogen storage in the body, the increase in fat oxidation due to exercise in a postabsorptive state may not be extrapolated to chronic effects. Accordingly, it is possible that the effects of postabsorptive exercise on expanding glycogen storage would eventually be counterbalanced by increased carbohydrate oxidation. Further studies are needed to examine the effects of regular postabsorptive exercise on fat metabolism.

Our studies examined participants with a variety of characteristics, including sexual differences, training status, exercise intensity and volume, and mode of exercise. However, the effects of body composition and age on 24 h fat oxidation have not been examined. Furthermore, while our studies have assumed a typical three-meal-a-day lifestyle, actual lifestyles are likely to be much more diverse. In the future, it may be necessary to study protocols that address diverse lifestyles.

## 6. Conclusions

Exercise in the postabsorptive state (i.e., overnight fasting) is the only spontaneous time of day that increases fat oxidation, which may be due to transient glycogen deficit. In the future, it will be necessary to investigate the synergistic effects of nutritional status and exercise as well as to determine which factor between postprandial/postabsorptive state or circadian rhythm is more prominent in the time-dependent effects of exercise.

## Data Availability

Not applicable.

## References

[1] Sports and Exercise. Bureau of Labor Statistics. United States Department of Labor. http://www.bls.gov/spotlight/2008/sports/.

[2] Survey on Time Use and Leisure Activities Statistics Bureau, Ministry of Internal Affairs and Communications. https://www.e-stat.go.jp/en/stat-search/files?page=1&toukei=00200533&tstat=000001158160.

[3] Heikura I.A., Stellingwerff T., Burke L.M. (2018). Self-reported periodization of nutrition in elite female and male runners and race walkers. Front. Physiol..

[4] Piercy K.L., Troiano R.P., Ballard R.M., Carlson S.A., Fulton J.E., Galuska D.A., George S.M., Olson R.D. (2018). The physical activity guidelines for Americans. JAMA.

[5] Rhodes R.E., Janssen I., Bredin S.S.D., Warburton D.E.R., Bauman A. (2017). Physical activity: Health impact, prevalence, correlates and interventions. Psychol. Health.

[6] Bull F.C., Al-Ansari S.S., Biddle S., Borodulin K., Buman M.P., Cardon G., Carty C., Chaput J.P., Chastin S., Chou R. (2020). World Health Organization 2020 guidelines on physical activity and sedentary behavior. Br. J. Sport. Med..

[7] US Department of Health and Human Services (2018). Physical Activity Guidelines for Americans.

[8] Ishay Y., Kolben Y., Kessler A., Ilan Y. (2021). Role of circadian rhythm and autonomic nervous system in liver function: A hypothetical basis for improving the management of hepatic encephalopathy. Am. J. Physiol..

[9] Gabriel B.M., Zierath J.R. (2019). Circadian rhythms and exercise—Re-setting the clock in metabolic disease. Nat. Rev. Endocrinol..

[10] Kim H.K., Radak Z., Takahashi M., Inami T., Shibata S. (2022). Chrono-exercise: Time-of-day-dependent physiological responses to exercise. Sport. Med. Health Sci..

[11] Bachman J.J., Deitrick R.W., Hillman A.R. (2016). Exercising in the fasted state reduced 24-hour energy intake in active male adults. J. Nutr. Metab..

[12] Tanaka Y., Ogata H., Kayaba M., Ando A., Park I., Yajima K., Araki A., Suzuki C., Osumi H., Zhang S. (2020). Effect of a single bout of exercise on clock gene expression in human leukocyte. J. Appl. Physiol..

[13] Yamanaka Y., Hashimoto S., Takasu N.N., Tanahashi U., Nishide S., Honma S., Honma K. (2015). Morning and evening physical exercise differentially regulate the autonomic nervous system during nocturnal sleep in humans. Am. J. Physiol..

[14] Vieira A.F., Costa R.R., Macedo R.C.O., Coconcelli L., Kruel L.F.M. (2016). Effects of aerobic exercise performed in fasted v. fed state on fat and carbohydrate metabolism in adults: A systematic review and meta-analysis. Br. J. Nutr..

[15] Blankenship J.M., Rosenberg R.C., Rynders C.A., Melanson E.L., Catenacci V.A., Creasy S.A. (2021). Examining the role of exercise timing in weight management: A review. Int. J. Sport. Med..

[16] Gillen J.B., Percival M.E., Ludzki A., Tarnopolsky M.A., Gibala M.J. (2013). Interval training in the fed or fasted state improves body composition and muscle oxidative capacity in overweight women. Obesity.

[17] Nybo L., Pedersen K., Christensen B., Aagaard P., Brandt N., Kiens B. (2009). Impact of carbohydrate supplementation during endurance training on glycogen storage and performance. Acta Physiol..

[18] Schoenfeld B.J., Aragon A.A., Wilborn C.D., Krieger J.W., Sonmez G.T. (2014). Body composition changes associated with fasted versus non-fasted aerobic exercise. J. Int. Soc. Sport. Nutr..

[19] Chtourou H., Souissi N. (2012). The effect of training at a specific time of day: A review. J. Strength Cond. Res..

[20] Van Proeyen K., Szlufcik K., Nielens H., Ramaekers M., Hespel P. (2011). Beneficial metabolic adaptations due to endurance exercise training in the fasted state. J. Appl. Physiol..

[21] Mancilla R., Krook A., Schrauwen P., Hesselink M.K.C. (2020). Diurnal regulation of peripheral glucose metabolism: Potential effects of exercise timing. Obesity.

[22] Terada T., Wilson B.J., Myette-Cote E., Kuzik N., Bell G.J., McCarggar L.J., Boule N.G. (2016). Targeting specific interstitial glycemic parameters with highintensity interval exercise and fasted-state exercise in type 2 diabetes. Metabolism.

[23] Romijn J.A., Coyle E.F., Sidossis L.S., Gastaldelli A., Horowitz J.F., Endert E., Wolfe R.R. (1993). Regulation of endogenous fat and carbohydrate metabolism in relation to exercise intensity and duration. Am. J. Physiol..

[24] Amaro-Gahete F.J., Jurado-Fasoli L., Trivino A.R., Sanchez-Delgado G., De-la-O A., Helge J.W., Ruiz J.R. (2019). Diurnal variation of maximal fat-oxidation rate in trained male athletes. Int. J. Sport Physiol. Perform..

[25] Sharma P., Agarwal M. (2022). Diurnal variation of fat oxidation rate and energy expenditure in an acute bout of endurance exercise by young healthy males. J. Fam. Med. Prim. Care.

[26] Kim H.K., Konishi M., Takahashi M., Tabata H., Endo N., Numao S., Lee S.K., Kim Y.H., Suzuki K., Sakamoto S. (2015). Effects of acute endurance exercise performed in the morning and evening on inflammatory cytokine and metabolic hormone responses. PLoS ONE.

[27] Aoyama S., Shibata S. (2020). Time-of-day-dependent physiological responses to meal and exercise. Front. Physiol..

[28] Maughan R.J., Fallah J., Coyle E.F. (2010). The effects of fasting on metabolism and performance. Br. J. Sport. Med..

[29] Horowitz J.F., Mora-Rodriguez R., Byerley L.O., Coyle E.F. (1997). Lipolytic suppression following carbohydrate ingestion limits fat oxidation during exercise. Am. J. Physiol..

[30] Bergman B.C., Brooks G.A. (1999). Respiratory gas-exchange ratios during graded exercise in fed and fasted trained and untrained men. J. Appl. Physiol..

[31] Bielinski Y., Schutz Y., Jequier E. (1985). Energy metabolism during the postexercise recovery in man. Am. J. Clin. Nutr..

[32] Bahr R., Sejersted O.M. (1991). Effect of intensity of exercise on excess postexercise O2 consumption. Metabolism.

[33] Kuo C.C., Fattor J.A., Henderson G.C., Brooks G.A. (2005). Lipid oxidation in fit young adults during postexercise recovery. J. Appl. Physiol..

[34] Børsheim E., Bahr R. (2003). Effect of exercise intensity, duration and mode on post-exercise oxygen consumption. Sport. Med..

[35] Gaesser C.A., Brooks G.A. (1984). Metabolic basis of post-exercise oxygen consumption: A review. Med. Sci. Sport. Exerc..

[36] Iwayama K., Miyashita M., Tokuyama K. (2008). Changes in substrate oxidation persist overnight after a marathon race. Jpn. J. Phys. Fit. Sport. Med..

[37] Chen K., Smith S., Ravussin E., Krakoff J., Plasqui G., Tanaka S., Murgatroyd P., Brychta R., Bock C., Carnero E. (2020). Room Indirect Calorimetry Operating and Reporting Standards (RICORS 1.0): A guide to conducting and reporting human whole-room calorimeter studies. Obesity.

[38] Tokuyama K., Ogata H., Katayose Y., Satoh M. (2009). Algorithm for transient response of whole body indirect calorimeter: Deconvolution with a regularization parameter. J. Appl. Physiol..

[39] Melanson E.L., Sharp T.A., Seagle H.M., Horton T.J., Donahoo W.T., Grunwald G.K., Hamilton J.T., Hill J.O. (2002). Effect of exercise intensity on 24-h energy expenditure and nutrient oxidation. J. Appl. Physiol..

[40] Melanson E.L., Gozansky W.S., Barry D.W., Maclean P.S., Grunwald G.K., Hill J.O. (2009). When energy balance is maintained, exercise does not induce negative fat balance in lean sedentary, obese sedentary, or lean endurance-trained individuals. J. Appl. Physiol..

[41] Treuth M.S., Hunter G.R., Williams M. (1996). Effects of exercise intensity on 24-h energy expenditure and substrate oxidation. Med. Sci. Sport. Exerc..

[42] Saris W.H.M., Schrauwen P. (2004). Substrate oxidation differences between high- and low-intensity exercise are compensated over 24 hours in obese men. Int. J. Obes..

[43] Melanson E.L., Cornier M.A., Bessesen D.H., Grunwald G.K., MacLean P.S., Hill J.O. 24 H Metabolic Responses to Low- and High-Intensity Exercise in Lean and Obese Humans. Proceedings of the 2005 NAASO Annual Scientific Meeting.

[44] Melanson E.L., Donahoo W.T., Grunwald G.K., Schwartz R. (2007). Changes in 24-h substrate oxidation in older and younger men in response to exercise. J. Appl. Physiol..

[45] Dionne I., Van Vugt S., Tremblay A. (1999). Postexercise macronutrient oxidation: A factor dependent on postexercise macronutrient intake. Am. J. Clin. Nutr..

[46] Iwayama K., Kurihara R., Nabekura Y., Kawabuchi R., Park I., Kobayashi M., Ogata H., Kayaba M., Satoh M., Tokuyama K. (2015). Exercise increases 24-h fat oxidation only when it is performed before breakfast. EBioMed.

[47] Shimada K., Yamamoto Y., Iwayama K., Nakamura K., Yamaguchi S., Hibi M., Nabekura Y., Tokuyama K. (2013). Effects of post-absorptive and postprandial exercise on 24 h fat oxidation. Metabolism.

[48] Iwayama K., Kawabuchi R., Park I., Kurihara R., Kyobayashi M., Hibi M., Oishi S., Yasunaga K., Ogata H., Nabekura Y. (2015). Transient energy deficit induced by exercise increases 24-h fat oxidation in young trained men. J. Appl. Physiol..

[49] Iwayama K., Ogawa A., Tanaka Y., Yajima K., Park I., Ando A., Ogata H., Kayaba M., Zhang S., Tanji F. (2020). Effects of exercise before breakfast on plasma free fatty acid profile and 24-h fat oxidation. Metabol. Open.

[50] Tanaka Y., Ogata H., Park I., Ando A., Ishihara A., Kayaba M., Yajima K., Suzuki C., Araki A., Osumi H. (2021). Effect of a single bout of morning or afternoon exercise on glucose fluctuation in young healthy men. Physiol. Rep..

[51] Iwayama K., Kawabuchi R., Nabekura Y., Kurihara R., Park I., Kobayashi M., Ogata H., Kayaba M., Omi N., Satoh M. (2017). Exercise before breakfast increases 24-h fat oxidation in female subjects. PLoS ONE.

[52] Aarts E., Verhage M., Veenvliet J.V., Dolan C.V., van der Sluis S. (2014). A solution to dependency: Using multilevel analysis to accommo-date nested data. Nat. Neurosc..

[53] Rui L. (2014). Energy metabolism in the liver. Compr. Physiol..

[54] Goldstein I., Hager G.L. (2015). Transcriptional and chromatin regulation during fasting-the genomic era. Trends Endocrinol. Metab..

[55] Izumida Y., Yahagi N., Takeuchi Y., Nishi M., Shikama A., Takarada A., Masuda Y., Kubota M., Matsuzaka T., Nakagawa Y. (2013). Glycogen shortage during fasting triggers liver–brain–adipose neurocircuitry to facilitate fat utilization. Nat. Commun..

[56] Philp A., Hargreaves M., Baar K. (2012). More than a store: Regulatory roles for glycogen in skeletal muscle adaptation to exercise. Am. J. Physiol..

[57] Midia M., Odedra D., Shuster A., Midia R., Muir J. (2019). Predictors of bleeding complications following percutaneous image-guided liver biopsy: A scoping review. Fiagn. Interv. Radiol..

[58] Takahashi H., Kamei A., Osawa T., Kawahara T., Takizawa O., Maruyama K. (2015). ^13^C MRS reveals a small diurnal variation in the glycogen content of human thigh muscle. NMR Biomed..

[59] Iwayama K., Onishi T., Maruyama K., Takahashi H. (2020). Diurnal variation in the glycogen content of the human liver using ^13^C MRS. NMR Biomed..

[60] Iwayama K., Tanabe Y., Tanji F., Ohnishi T., Takahashi H. (2021). Diurnal variations in muscle and liver glycogen differ depending on the timing of exercise. J. Physiol. Sci..

[61] Arciero P.J., Ives S.J., Mohr A.E., Robinson N., Escudero D., Robinson J., Rose K., Minicucci O., O’Brien G., Curran K. (2022). Morning exercise reduces abdominal fat and blood pressure in women; evening exercise increases muscular performance in women and lowers blood pressure in men. Front. Physiol..

[62] De Bock K., Derave W., Eijnde B.O., Hesselink M.K., Koninckx E., Rose A.J., Schrauwen P., Bonen A., Richter E.A., Hespel P. (2008). Effect of training in the fasted state on metabolic responses during exercise with carbohydrate intake. J. Appl. Physiol..

[63] Flatt J.P. (1988). Importance of nutrient balance in body weight regulation. Diabetes Metab. Rev..

